# Spectral CT - a new supplementary method for preoperative assessment of pathological grades of esophageal squamous cell carcinoma

**DOI:** 10.1186/s12880-023-01068-5

**Published:** 2023-08-23

**Authors:** Yi Wang, Weizhong Tian, Shuangfeng Tian, Liang He, Jianguo Xia, Ji Zhang

**Affiliations:** Department of Radiology, Taizhou People’s Hospital, NO.366 Taihu Road, Yiyaogaoxin District, Taizhou, 225300 Jiangsu China

**Keywords:** Esophageal squamous cell carcinoma, Spectral CT, Pathological grade

## Abstract

**Background:**

Spectral CT imaging parameters have been reported to be useful in the differentiation of pathological grades in different malignancies. This study aims to investigate the value of spectral CT in the quantitative assessment of esophageal squamous cell carcinoma (ESCC) with different degrees of differentiation.

**Methods:**

There were 191 patients with proven ESCC who underwent enhanced spectral CT from June 2018 to March 2020 retrospectively enrolled. These patients were divided into three groups based on pathological results: well differentiated ESCC, moderately differentiated ESCC, and poorly differentiated ESCC. Virtual monoenergetic 40 keV-equivalent image (VMI_40keV_), iodine concentration (IC), water concentration (WC), effective atomic number (Eff-Z), and the slope of the spectral curve(λ_HU_) of the arterial phase (AP) and venous phase (VP) were measured or calculated. The quantitative parameters of the three groups were compared by using one-way ANOVA and pairwise comparisons were performed with LSD. Receiver operating characteristic (ROC) analysis was used to evaluate the diagnostic performance of these parameters in poorly differentiated groups and non-poorly differentiated groups.

**Results:**

There were significant differences in VMI_40keV_, IC, Eff-Z, and λ_HU_ in AP and VP among the three groups (all *p* < 0.05) except for WC (*p* > 0.05). The VMI_40keV_, IC, Eff-Z, and λ_HU_ in the poorly differentiated group were significantly higher than those in the other groups both in AP and VP (all *p* < 0.05). In the ROC analysis, IC performed the best in the identification of the poorly differentiated group and non-poorly differentiated group in VP (AUC = 0.729, Sensitivity = 0.829, and Specificity = 0.569 under the threshold of 21.08 mg/ml).

**Conclusions:**

Quantitative parameters of spectral CT could offer supplemental information for the preoperative differential diagnosis of ESCC with different degrees of differentiation.

## Introduction

 According to the global cancer statistics 2020, esophageal cancer ranks seventh in terms of incidence and sixth in mortality overall, the latter signifying that esophageal cancer is responsible for one in every 18 cancer deaths in 2020 [1]. The treatment strategies and prognosis evaluation for esophageal cancer are mainly determined by the clinical TNM stage and pathological TNM (pTNM) stage, the latter remains relevant for early-stage cancers and as an important staging and survival reference point [2]. The overall 5-year survival of patients with esophageal cancer ranges from 15 to 25%. Diagnoses made at earlier stages are associated with better outcomes than those made at later stages [3], the latter is more prone to lymph node metastasis and local recurrence as well as shorter survival [4]. In China, esophageal squamous cell carcinoma (ESCC) is the predominant pathological type of esophageal cancer [1]. A definitive diagnosis of esophageal lesions is based on histological examination; however, an invasive method is not always readily available, and local samples might not fully reflect the overall heterogeneity of the tumor. The degree of differentiation of esophageal cancer is an important component of pTNM staging of esophageal cancer, and well-differentiated esophageal cancer is less malignant and will be better treated compared to moderately and poorly differentiated esophageal cancer. Thus, an accurate and non-invasive method is urgently needed in evaluating the pathological grade of ESCC.

It is generally accepted that CT is an important modality for evaluating esophageal tumors. Conventional CT is often used to identify the location of primary lesions and distal metastases, but its role in determining pTNM is limited. Spectral CT has been widely used for qualitative and quantitative imaging of different malignancies and could provide more accurate and complete information on cancers for detection and prognoses evaluation. With the use of material decomposition techniques, one can obtain virtual monoenergetic images (VMI), iodine concentration (IC), water concentration (WC), effective atomic number (Eff-Z), or other material-specific information [5]. In recent years, spectral CT imaging parameters have been reported to be useful in the differentiation of pathological grades in different malignancies including glioma [6], pancreatic neuroendocrine neoplasms [7], gastric adenocarcinoma [8], ovarian tumours [9], non-small cell lung cancer [10], and clear cell renal cell carcinoma (ccRCC) [11]. However, to the best of our knowledge, spectral parameters have not been applied for the pathological grades of esophageal cancer so far.

Consequently, this study was conducted to explore the significance of quantitative assessment with several parameters derived from spectral CT in differentiating ESCC with different degrees of differentiation.

## Materials and methods

### Patient characteristics

This retrospective study was approved by the Ethics Committee of our hospital and all patients signed the informed consent. A total of 191 patients with histologically proven ESCC were enrolled in this study from June 2018 to March 2020. The inclusion criteria were as follows: (1) No contraindications to contrast-enhanced CT examination; (2) No radiation therapy or chemotherapy before surgery; (3) Within 1 week after spectral CT scan the lesions were resected; (4) Postoperative pathologic confirmation of ESCC. The main exclusion criteria were as follows: (1) Patients who were found to be allergic to iodine contrast agent before enhanced CT examination; (2) Patients who had already received radiation therapy or chemotherapy; (3) Images with poor quality due to artifacts.

### Spectral CT image acquisition

All inspections were conducted using a Revolution CT scanner (GE Healthcare, Milwaukie USA) with the spectral CT acquisition mode. The scan protocol included a 5 mm slice thickness, tube voltage of 70 and 140 kV with a fast kilovolt peak–switching technique, CT automatic exposure control (AEC) systems adjusting the tube current, and gantry speed of 0.5 s per rotation, and helical pitch of 0.992:1. The nonionic contrast agent Iohexol (China, Jiangsu Yangtze River Pharmaceutical Group) was used for the enhanced examination of patients, containing 300mgI/ml of iodine, the weight-dependent dose of 1.5 ml/kg, and an infusion rate of 3.0 ml/s. Scanning was done when the CT value of the aortic arch reached 100 HU, and the arterial phase (AP) and venous phase (VP) started 30 and 60 s, respectively, after the administration of contrast agents.

### Spectral CT image analysis

All spectral CT images were reconstructed with a slice thickness of 1.25 mm, and then the images were transferred to the workstation. Two radiologists with 6 and 20 years of experience in esophagus CT diagnosis measured and analyzed the imaging in a blinded and randomized manner respectively. A round or oval region of interest (ROI) was selected according to the size and location of the lesions to measure the virtual monoenergetic 40 keV-equivalent image (VMI_40keV_), IC, WC, and Eff-Z in both the AP and VP. To reduce measurement variation, ROIs were placed three times in the tumor area without distinguishable necrosis or hemorrhage, and the average of triplicate measurements was used as the final data value. The slope of the spectral curve (λ_HU_) was defined as the difference between the CT value at 40 keV and that at 70 keV divided by the energy difference(30 keV), and it was calculated as follows: λ_HU_ = [(CT(40 keV)-CT(70 keV)]/(70 − 40) [12].

### Pathological analysis

All tissues were obtained from surgical operation. Based on the 8th edition of AJCC classification [4], these patients were divided into three groups according to postoperative pathological results: well differentiated ESCC, moderately differentiated ESCC and poorly differentiated ESCC.

### Statistical analysis

Statistical analyses were conducted using SPSS26.0. Quantitative variables are expressed as mean ± standard deviation, and categorical variables are presented as frequencies (percentages). The differences in VMI_40keV_, IC, WC, Eff-Z, and λ_HU_ of the three groups were statistically analyzed using one-way ANOVA and pairwise comparison was performed with LSD. Receiver operating characteristic (ROC) analysis was conducted for each parameter to differentiate the poorly differentiated and non-poorly differentiated groups.

## Results

### Patient characteristics

A total of 191 patients (mean age 68.49 ± 6.78 years), 157 men (mean age 68.34 ± 7.16 years), and 34 women (mean age 69.18 ± 4.64 years) were included. Characteristics of our patients are summarized in Table 1.

**Table 1:** Patient characteristics

Group	n	Sex		age (y)	ROI (mm^2^)		Lymph node involvement
		F	M		AP	VP	
Well differentiated ESCC	27	0	27	67.0 ± 7.0	36 ~ 199	35 ~ 199	17
Moderately differentiated ESCC	82	20	62	66.4 ± 7.2	26 ~ 184	27 ~ 181	27
Poorly differentiated ESCC	82	14	68	71.1 ± 5.4	30 ~ 150	28 ~ 158	33

*ESCC *Esophageal squamous cell carcinoma, *ROI *Region of interest, *AP *Arterial phase, *VP *Venous phase.

### Quantitative spectral parameters comparison

There were significant differences in VMI_40keV_, IC, Eff-Z, and λ_HU_ in AP and VP among the three groups (all *p* < 0.05) except for WC (*p* > 0.05). The VMI_40keV_, IC, Eff-Z, and λ_HU_ in the poorly differentiated group were significantly higher than those in the other groups both in AP and VP (all *p* < 0.05; Table 2).

**Table 2:** Comparison of quantitative parameters among ESCC of different degrees of differentiation in AP and VP

Phase	Group	n	VMI_40keV_	IC (mg/ml)	WC (mg/ml)	Eff-Z	λ_HU_
AP	Well differentiated ESCC	27	137.85 ± 25.18	13.66 ± 3.13	1024.74 ± 7.19	8.43 ± 0.17	2.58 ± 0.59
	Moderately differentiated ESCC	82	162.81 ± 45.75	16.49 ± 5.29	1026.35 ± 5.92	8.58 ± 0.28	3.12 ± 1.00
	Poorly differentiated ESCC	82	172.18 ± 44.41	17.65 ± 5.13	1026.13 ± 8.60	8.64 ± 0.27	3.33 ± 1.00
	F Value		6.518	6.563	0.504	6.907	6.554
	*P* Value		0.002	0.002	0.605	0.001	0.002
VP	Well differentiated ESCC	27	192.66 ± 29.44	19.85 ± 3.59	1028.66 ± 5.80	8.76 ± 0.18	3.74 ± 0.68
	Moderately differentiated ESCC	82	210.98 ± 80.27	21.31 ± 4.58	1027.24 ± 9.21	8.84 ± 0.24	4.27 ± 2.46
	Poorly differentiated ESCC	82	234.88 ± 44.03	24.88 ± 5.15	1029.18 ± 7.53	9.01 ± 0.24	4.70 ± 0.97
	F Value		5.987	17.217	1.213	3.277	17.877
	*P* Value		0.003	0.000	0.300	0.040	0.000

Except for the F and P-values, data are reported as mean ± standard deviation.

*ESCC *Esophageal squamous cell carcinoma, *AP *Arterial phase, *VP *Venous phase, *VMI *Virtual monoenergetic image, *IC *Iodine concentration, *WC *Water concentration, *Eff-Z *Effective atomic number; λ_HU_, the slope of the spectral curve.

### Quantitative diagnostic value evaluation

**Fig. 1:**
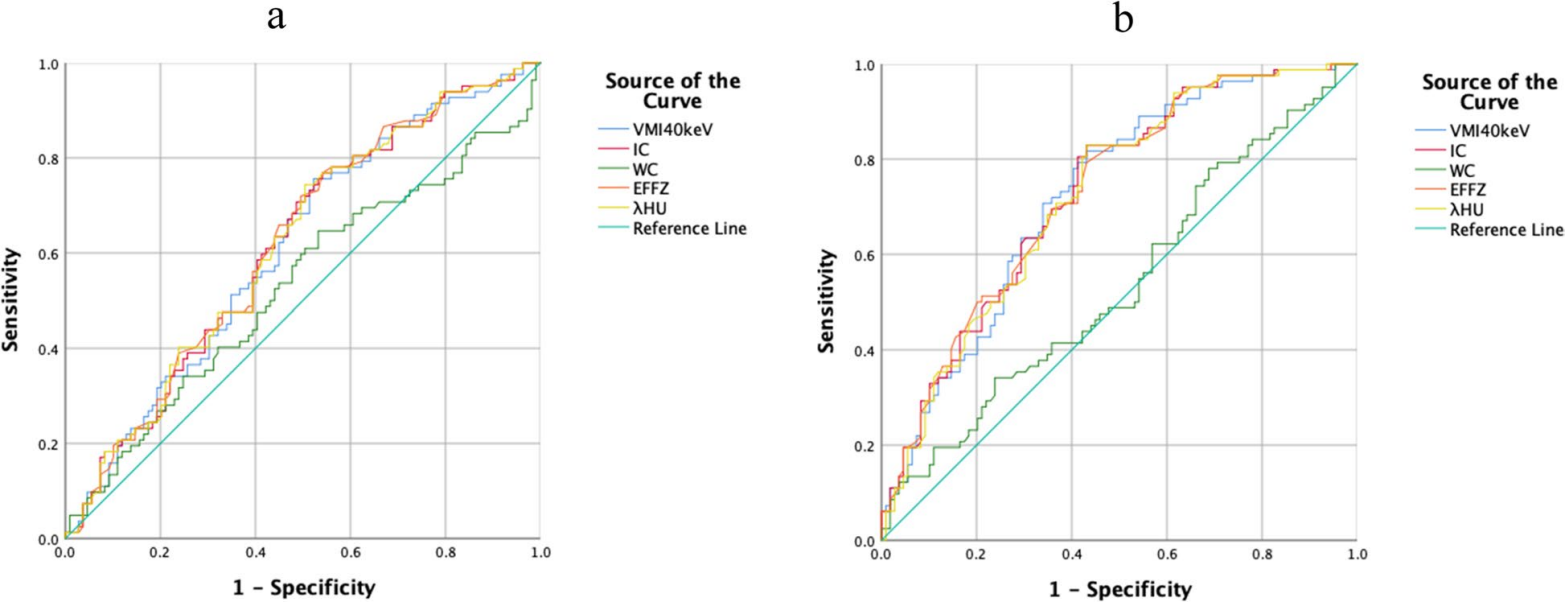
Receiver operating characteristic curves of each parameter for the differential diagnosis of the poorly differentiated group (82 cases) from the non-poorly differentiated group (109 cases) in the arterial phase and venous phase, respectively. **a** the arterial phase; **b** the venous phase. VMI, virtual monoenergetic image; IC, iodine concentration; WC, water concentration; Eff-Z, effective atomic number; λ_HU_, the slope of the spectral curve

ROC curves for all spectral CT parameters are shown in Fig. 1. Table 3 shows AUCs, thresholds, Sensitivities, and Specificities based on the ROC analysis.

**Table 3:** AUCs, thresholds, sensitivities, and specificities for distinguishing the poorly differentiated group from the non-poorly differentiated group in AP and VP

Parameter	AP				VP			
	AUC	Threshold value	Sensitivity	Specificity	AUC	Threshold value	Sensitivity	Specificity
VMI_40keV_	0.612	140.30	75.6%	47.7%	0.725	204.36	81.7%	56.9%
IC	0.616	13.70	76.8%	45.9%	0.729	21.08	82.9%	56.9%
Eff-Z	0.619	8.44	76.8%	45.9%	0.729	8.84	79.3%	56.9%
λ_HU_	0.617	2.64	74.4%	49.5%	0.723	3.97	82.9%	56.9%

*AUC *Area under the ROC curve, *AP *Arterial phase, *VP *Venous phase, *VMI *Virtual monoenergetic image, *IC *Iodine concentration, *Eff-Z *Effective atomic number; λ_HU_, the slope of the spectral curve

In the identification of poorly differentiated groups and non-poorly differentiated groups. VMI_40keV_, IC, Eff-Z, and λ_HU_ in VP showed an area under the ROC curve (AUC) of 0.725, 0.729, 0.729, and 0.723, respectively, while AP showed an AUC of 0.612, 0.616, 0.619, and 0.617, respectively. The highest sensitivity, specificity, and AUC were observed for IC in VP, the sensitivity of it in the identification between the poorly differentiated group and the non-poorly differentiated group was 82.9%, while the specificity was 56.9% under the threshold of 21.08 mg/ml.

## Discussion

**Fig. 2:**
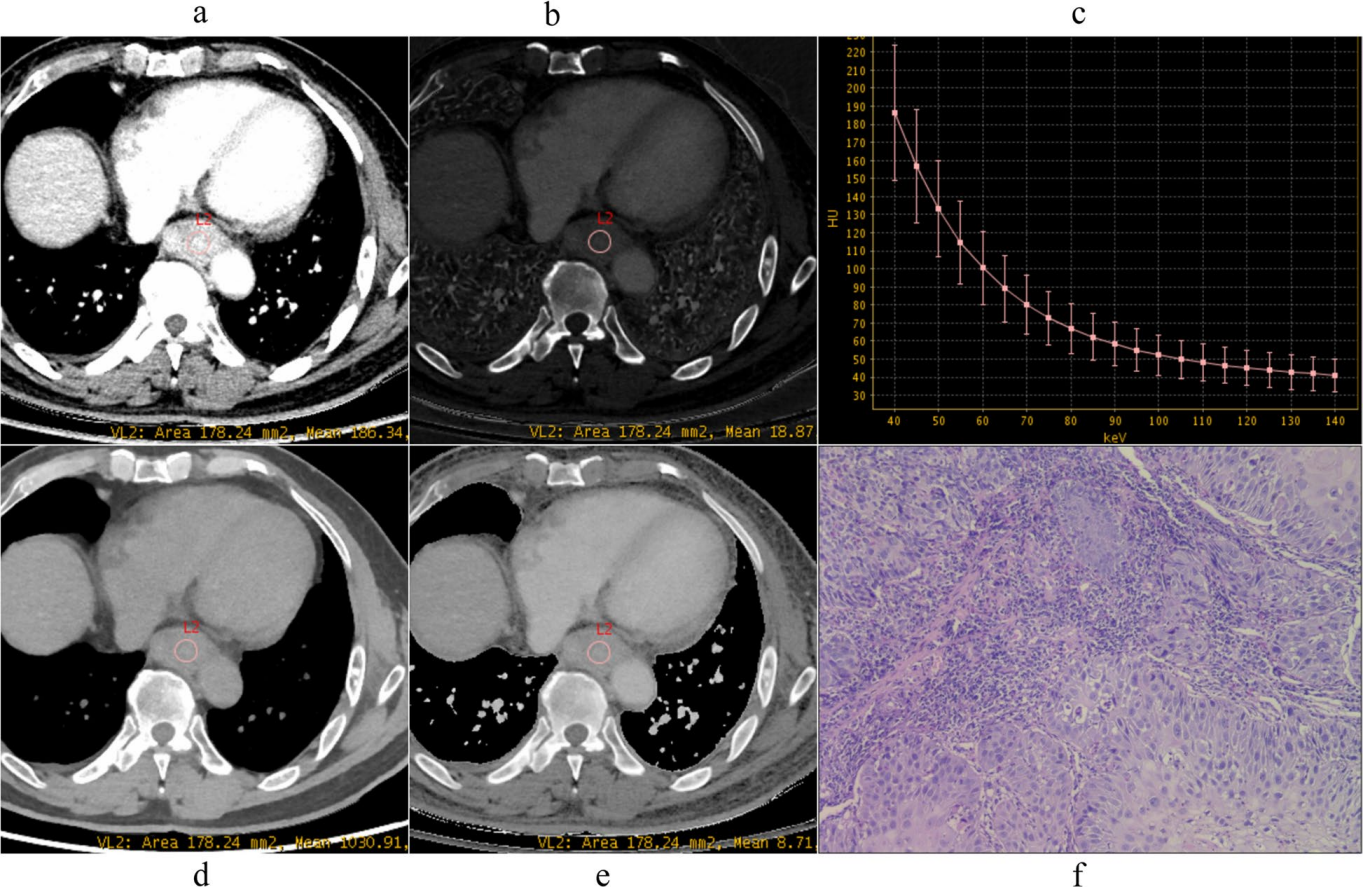
Spectral images in venous phase and Photomicrograph (original magnification, ×100) example of well differentiated ESCC (male, 71 years old). **a** VMI_40 keV_=185.88 HU, **b** IC = 18.88 mg/ml, **c** λ_HU_ = 3.57, **d** WC = 1029.58 mg/ml, **e** Eff-Z = 8.71, **f** Photomicrograph (original magnification, ×100). VMI, virtual monoenergetic image; IC, iodine concentration; WC, water concentration; λ_HU_, the slope of the spectral curve; Eff-Z, effective atomic number

**Fig. 3:**
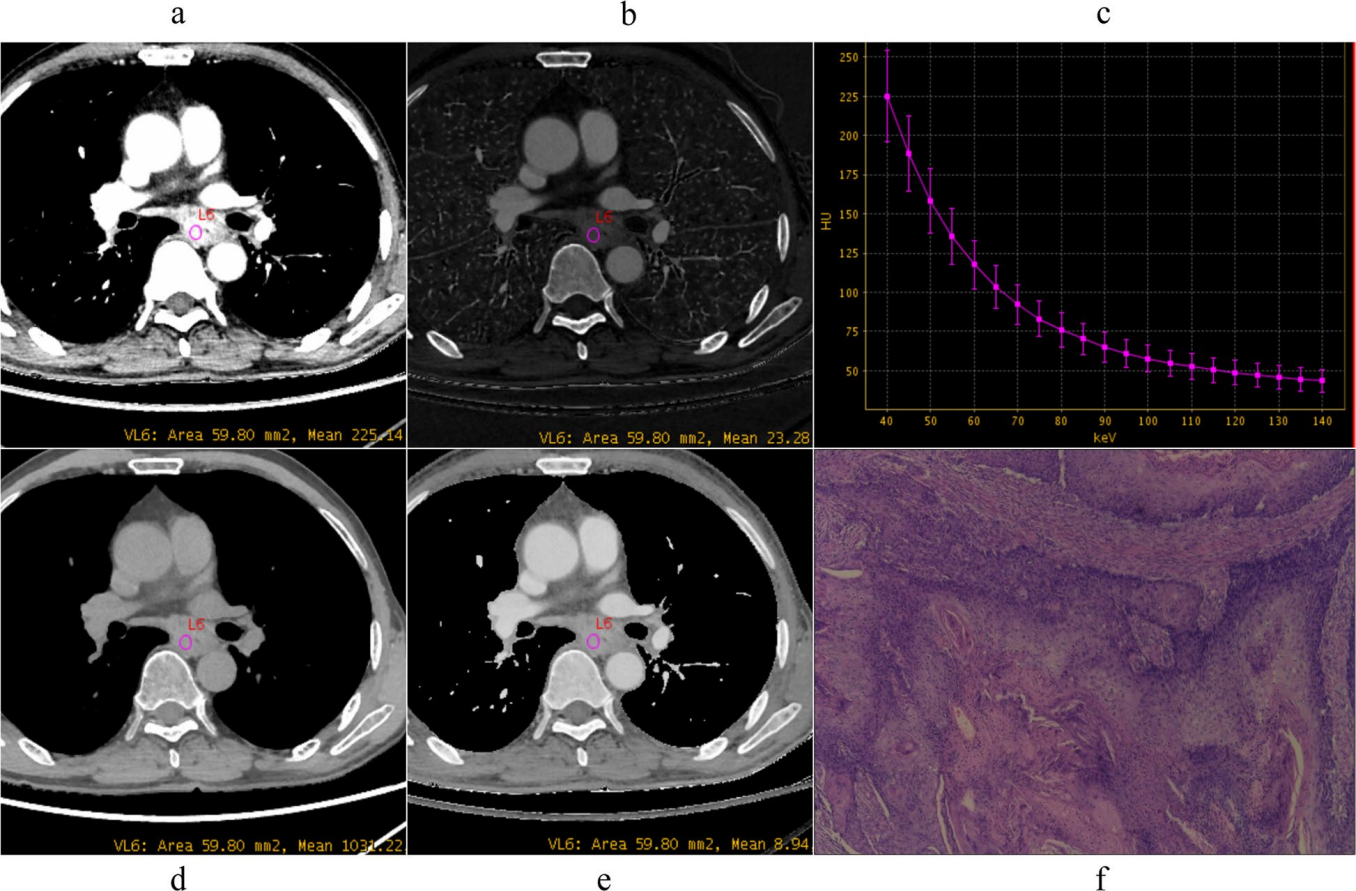
Spectral images in venous phase and Photomicrograph (original magnification, ×100) example of moderately differentiated ESCC (male, 68 years old). **a** VMI_40 keV_=225.76 HU, **b** IC = 23.10 mg/ml, **c** λ_HU_ = 4.36, **d** WC = 1034.81 mg/ml, **e** Eff-Z = 8.93, **f** photomicrograph (original magnification, ×100). VMI, virtual monoenergetic image; IC, iodine concentration; WC, water concentration; λ_HU_, the slope of the spectral curve; Eff-Z, effective atomic number

**Fig. 4:**
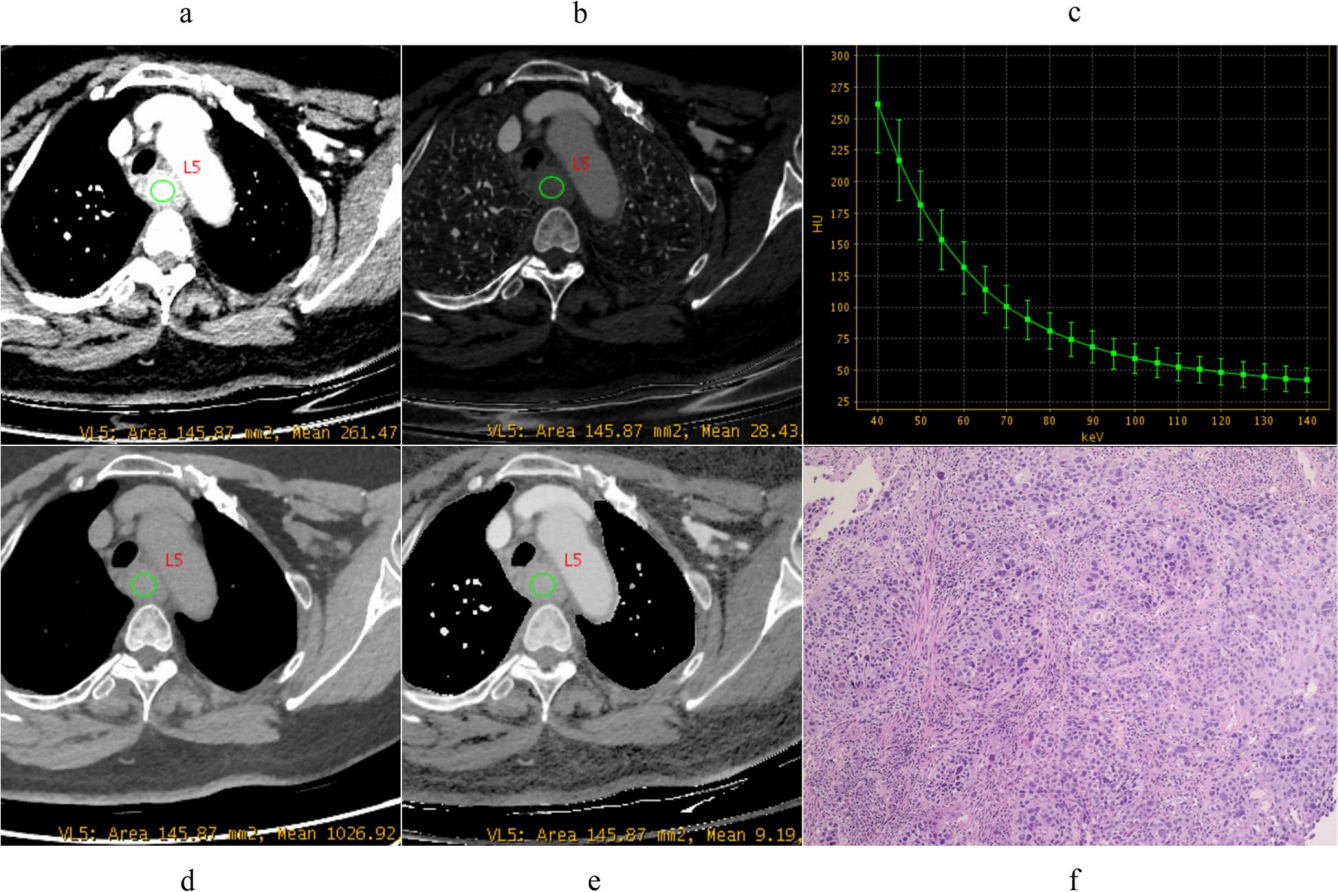
Spectral images in venous phase and Photomicrograph (original magnification, ×100) example of poorly moderately differentiated ESCC (female, 64 years old). **a** VMI_40 keV_=261.35 HU, **b** IC = 28.52 mg/ml, **c** λ_HU_ = 5.36, d: WC = 1026.40 mg/ml, **e** Eff-Z = 9.20, **f** Photomicrograph (original magnification, ×100). VMI, virtual monoenergetic image; IC, iodine concentration; WC, water concentration; λ_HU_, the slope of the spectral curve; Eff-Z, effective atomic number

The pathological differentiation grade of ESCC influences the prognosis, as tumor grade increases, it is more likely to have a poorer prognosis and an elevated risk of death [10]. Our results showed that quantitative parameters derived from spectral CT, including VMI_40keV_, IC, Eff-Z, and λ_HU_ both in AP and VP, could be used to distinguish pathological grades of ESCC, as shown in Figs. 2, 3 and 4.

Spectral CT greatly reduces beam-hardening artifacts and generates VMIs with more accurate CT attenuation numbers at every energy level [13]. VMI_40keV_ reconstructions provided higher image quality due to a higher lesion to background attenuation ratio, i.e., higher contrast [14, 15]; Thus, we selected the 40 keV image for this study. In the present study, VMI_40keV_ in the AP and VP was significantly different among the three groups, which concurs with a previous study [13].

The distribution of iodine in the tissue is strongly correlated with local blood volume and vascular density [16]. Several studies have shown that IC correlates well with higher blood flow and vascularization [14, 17–22]. In our study, IC in the AP and VP was significantly different among the three groups, as tumor grade increased, it also increased. To be more precise, IC in the well differentiated group was the lowest while in the poorly differentiated ESCC group was the highest, signifying that the iodine uptake and vascularity of the low-grade ESCCs are lower than that of high-grade ESCCs.

Additionally, Eff-Z, another quantitative index for different materials which represents the composite atom for a compound or mixture of various materials and is important to predict how x-rays interact with a substance [7], was analyzed in the current study. Previous studies have indicated that Eff-Z could depict lesion characterization and could be used to differentiate tumors [16, 23–25]. In our study, the same conclusions were reached. There were significant differences in Eff-Z among the three groups in both AP and VP. Eff-Z in high-grade ESCCs was higher than in low-grade ESCCs, indicative of the feasibility of Eff-Z as a differentiating factor for ESCCs with different degrees of differentiation.

Regarding the λ_HU_ of ESCC, λ_HU_ and the tumor pathological grade showed a significant association during both phases, and tumors with a lower grade had lower λ_HU_. The spectral curve reflects different lesions or tissues that absorb X-rays at different rates [26]. Thus, our study indicated that with the increase in pathological grade of ESCC, the local enhancement and the iodine contrast agent of local lesions increased, which concurred with previous studies [9–11].

With regards to WC, it is not dependent on photon energy and is less affected by the beam hardening effect, unlike the CT attenuation number, and therefore it is a more reliable parameter in tumor characterization [27]. However, inconsistent with previous studies [9, 28], our study demonstrated that WC was not significantly different among the three groups, which was useless for differentiating pathological grades of ESCC. This may be related to the small sample size of this study, which needs further study and verification in the future.

The prognosis of low-grade ESCC is better than high-grade ESCC, it will be of great value if we can distinguish them from each other on spectral CT. Thus, in our study ROC was generated to evaluate the diagnostic performance of spectral parameters to distinguish between the poorly differentiated group and the non-poorly differentiated group. The best diagnostic performance was found for IC in VP using a threshold value of 21.08 mg/ml, which resulted in a sensitivity, specificity, and AUC of 0.829, 0.569, and 0.729, respectively, also suggesting that tumor cells were metabolized vigorously and blood supply was abundant [28]. The diagnostic efficiency of spectral parameters was lower in the AP than in the VP, which is consistent with a previous study [12]. This may be because the contrast medium can fill the microvessels and penetrates the basement membrane into the intercellular space in the VP [24]. Therefore, the spectral parameters of the VP can better reflect the histological characteristics of the tumor.

This study still has some limitations. Firstly, the study included a relatively small number of ESCC patients. Secondly, there is an inherent selection bias due to this study’s retrospective nature. Thirdly, our study was based on the patients with ESCC, therefore, it can’t be applied to other esophageal tumors, such as esophageal adenocarcinoma (EAC). Moreover, our study primarily focused on esophageal lesions without lymph node involvement. However, we encourage subsequent studies addressing these problems.

## Conclusions

In conclusion, parameters derived from spectral CT imaging could offer supplemental information to differentiate ESCC with different degrees of differentiation, which is particularly important in patients who can’t receive biopsy or surgery and might be a useful technology to help guide appropriate clinical diagnosis and prognosis.

## Data Availability

The datasets supporting the conclusions of this article are included within the article and its additional files.
